# Factors Influencing Clinicians’ Use of Hospital Information Systems for Infection Prevention and Control: Cross-Sectional Study Based on the Extended DeLone and McLean Model

**DOI:** 10.2196/44900

**Published:** 2023-06-22

**Authors:** Feiyang Zheng, Kang Wang, Qianning Wang, Tiantian Yu, Lu Wang, Xinping Zhang, Xiang Wu, Qian Zhou, Li Tan

**Affiliations:** 1 School of Medicine and Health Management Tongji Medical College of Huazhong University of Science and Technology Wuhan China; 2 School of Nursing Hong Kong Polytechnic University Kowloon Hong Kong; 3 Department of Hospital Infection Management Wuhan Children’s Hospital (Wuhan Maternal and Child Healthcare Hospital), Tongji Medical College Huazhong University of Science and Technology Wuhan China; 4 Tongji Hospital Tongji Medical College of Huazhong University of Science & Technology Wuhan China

**Keywords:** hospital infection prevention and control, information system, information systems success model

## Abstract

**Background:**

Healthcare-associated infections have become a serious public health problem. Various types of information systems have begun to be applied in hospital infection prevention and control (IPC) practice. Clinicians are the key users of these systems, but few studies have assessed the use of infection prevention and control information systems (IPCISs) from their perspective.

**Objective:**

This study aimed to (1) apply the extended DeLone and McLean Information Systems Success model (D&M model) that incorporates IPC culture to examine how technical factors like information quality, system quality, and service quality, as well as organizational culture factors affect clinicians’ use intention, satisfaction, and perceived net benefits, and (2) identify which factors are the most important for clinicians’ use intention.

**Methods:**

A total of 12,317 clinicians from secondary and tertiary hospitals were surveyed online. Data were analyzed using partial least squares-structural equation modeling and the importance-performance matrix analysis.

**Results:**

Among the technical factors, system quality (β=.089-.252; *P*<.001), information quality (β=.294-.102; *P*<.001), and service quality (β=.126-.411; *P*<.001) were significantly related to user satisfaction (*R*^2^=0.833), use intention (*R*^2^=0.821), and perceived net benefits (communication benefits [*R*^2^=0.676], decision-making benefits [*R*^2^=0.624], and organizational benefits [*R*^2^=0.656]). IPC culture had an effect on use intention (β=.059; *P*<.001), and it also indirectly affected perceived net benefits (β=.461-.474; *P*<.001). In the importance-performance matrix analysis, the attributes of service quality (providing user training) and information quality (readability) were present in the fourth quadrant, indicating their high importance and low performance.

**Conclusions:**

This study provides valuable insights into IPCIS usage among clinicians from the perspectives of technology and organization culture factors. It found that technical factors (system quality, information quality, and service quality) and hospital IPC culture have an impact on the successful use of IPCISs after evaluating the application of IPCISs based on the extended D&M model. Furthermore, service quality and information quality showed higher importance and lower performance for use intention. These findings provide empirical evidence and specific practical directions for further improving the construction of IPCISs.

## Introduction

Healthcare-associated infections (HAIs) have become a significant public health concern, posing a threat to the well-being of people and resulting in the spread of drug-resistant bacteria, an increase in unnecessary fatalities, and additional health care expenses [1]. The World Health Organization reports that 7% of hospitalized patients in high-income countries and 15% in low- and middle-income countries will be infected with at least one HAI, of which 1% will die [2]. Fortunately, it was estimated that 50% or more of HAIs could be prevented through continuous infection prevention and control (IPC) interventions, such as antimicrobial monitoring and surveillance [3]. Evidence indicates that monitoring and surveillance based on information systems can effectively reduce HAIs among hospitalized patients [4-6], and this kind of IPC intervention has been listed as a core component of an effective IPC plan by the World Health Organization [7]. Despite the potential benefits of information technology support in IPC practices, there are still several shortcomings that need to be addressed. First, there is a lack of timely response and user-friendly information systems at the system quality level, with system failure being the most common problem [8,9]. Second, at the level of information quality, the data are scattered across multiple systems, resulting in low-quality information lacking integrity and readability that cannot support analysis and decision-making [10-12]. Most hospitals lack service support for the user, such as providing user training [13]. Additionally, a lack of awareness and attention from medical staff and management on the application of information systems has resulted in obstacles such as “clinicians fighting against new technologies” or “it takes longer for clinicians to use new technologies to complete tasks” [14]. In summary, current IPC practices based on information technology support still have several deficiencies that need to be addressed.

The information system is an integrated system of components to collect, store, and process data. The data are used to provide information and contribute knowledge to support operations, management, and decision-making [15]. In this study, infection prevention and control information systems (IPCISs) refer to systems involved in acquiring, providing, and processing hospital infection–related data by clinicians when participating in IPC tasks, including hospital infection monitoring systems, early warning systems, doctor workstation systems, electronic medical record systems, etc. Research on the application of IPCISs mainly involves automatic cluster alert systems [16], the hospital infection control automatic monitoring toolkit [11,17,18], antimicrobial resistance monitoring systems [6,19,20], clinical decision support systems [19], electronic hand hygiene monitoring information systems [21,22], etc. Nevertheless, evaluations of IPCISs primarily focus on the systems’ design and deployment scheme, and their objective efficiency [5,23-25]. The technical evaluation of various types of IPCISs remains highly significant due to their shared similar information technology principles and basic functional modules, despite the existence of numerous IPCIS variants. However, there are few large-scale comprehensive evaluation studies on the application of IPCISs. The theoretical support of the evaluation is weak, and there is a lack of empirical evidence, especially evaluation evidence from direct user clinicians [9,26].

The DeLone and McLean Information Systems Success model (D&M model) is one of the widely used models for evaluating the successful application of information systems, providing classification metrics for information system success, as well as models of temporal and causal relationships between categories [27,28]. The D&M model has been extensively applied in finance [29,30], education [31-33], and business management [34,35]. More recently, the model has also been used in the medical and health fields. For example, Van Der Meijden et al summarized the success determinants of clinical information systems for inpatients based on the D&M model [36]. Yu et al constructed an information system success model for electronic prescribing from the perspective of doctors and pharmacists [37]. Other scholars have used the D&M model to evaluate electronic medical record systems and computerized physician order entry systems [38]. Most existing D&M models only evaluate information system success at the technical level, ignoring the impact of the social characteristics within organizations. However, social technology theory has shown that the application and effectiveness of information technology are also influenced by the social characteristics of organizations [39,40]. In the field of IPC, a critical social feature is the culture of IPC, which refers to “the increasingly stable common values and ideas gradually formed by improving the prevention and control of nosocomial infection.” The shared values and awareness determine how organization members devote their attention and actions for minimizing patient harm from HAIs [41]. These actions include the active use of information technology to report and predict situations such as drug-resistant infections. Therefore, the IPCIS evaluation also needs to consider the impact of the hospital IPC culture.

This study focused on evaluating clinicians’ use of IPCISs for several reasons. First, policy requirements from the Association for Professionals in Infection Control and Epidemiology (APIC) highlight the need to strengthen the information ability of relevant personnel for prevention and control, including monitoring information technology and using electronic medical records and system data [42]. Second, clinicians’ main tasks involved in IPC require technical support from information systems, such as monitoring drug-resistant bacteria trends in hospitals, submitting patients for pathogenic examination and drug sensitivity tests, adjusting antibiotic prescriptions, and communicating with other medical staff about drug resistance–related issues [43]. Lastly, there is a research gap in evaluating IPCIS use from clinicians’ perspectives, which needs to be addressed to improve their work efficiency and service quality [44].

Motivated by the analyses above, this study proposed an extended D&M model as the basis of the theoretical framework that adds cultural factors to construct an IPCIS evaluation model of clinicians’ perceptions in the context of IPC. According to our developed model, this study assessed the success of IPCISs in a survey of 12,317 clinicians from secondary and tertiary hospitals.

## Methods

### Research Model and Hypothesis

#### Research Model

**Figure 1:**
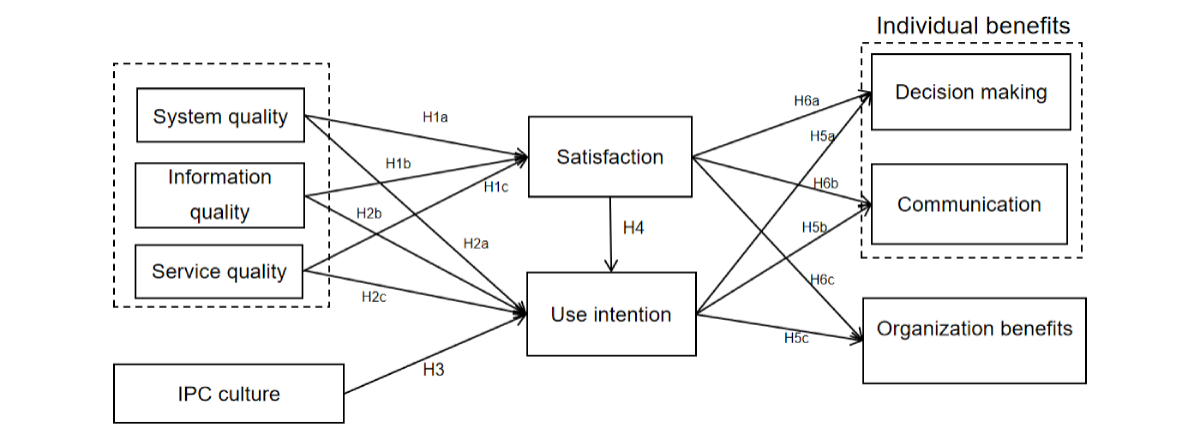
Proposed research model. The hypotheses are as follows: H1: Technical factors (system quality [H1a], information quality [H1b], and service quality [H1c]) have a positive impact on users’ satisfaction; H2: Technical factors (system quality [H2a], information quality [H2b], and service quality [H2c]) have a positive impact on users’ use intention; H3: The culture of IPC has a positive impact on the use of IPCISs; H4: User satisfaction has a positive impact on the intention to use the system; H5: Use intention has a positive impact on individual benefits (decision-making [H5a] and communication [H5b]) and organizational benefits (H5c); H6: User satisfaction has a positive impact on individual benefits (decision-making [H6a] and communication [H6b]) and organizational benefits (H6c). IPC: infection prevention and control; IPCIS: infection prevention and control information system.

This study designed 8 constructs based on the D&M model: system quality [27,44], information quality [44], service quality [28,44], use intention [44], user satisfaction [44], personal communication benefits [45], personal decision-making benefits [45], and organizational benefits [44], and 1 construct based on the perspective of organization: IPC culture [46]. The definition and measurement dimensions of each construct are shown in Table 1. The research model is shown in Figure 1.

**Table 1:** The construct definition and measurement items of the extended DeLone and McLean Information Systems Success model.

Construct	Definition	Measurement items	Source
System quality (SQ)	The desirable characteristics of information systems, such as usability, responsiveness, and user friendliness.	SQ1: Using the information system to submit requests for inspection, obtain antimicrobial susceptibility testing reports, and prescribe antibiotics is easy. SQ2: The response of the information system is very fast (such as cross-section/system operation without lag, etc). SQ3: The user interface design of the information system is clear and reasonable, and the functions are complete.	[27,44]
Information quality (IQ)	The desirable characteristics of information system outputs, such as integrity, real time, and legibility.	IQ1: The information system can provide me with the complete data information I need. IQ2: The information system can provide me with the real-time data information I need. IQ3: The information system provides information fields and reports that are displayed in an organized and easy-to-read manner.	[44]
Service quality (FQ)	The quality of the support that system users receive from the information system department and IT support personnel, such as responsiveness, empathy of the staff, and technical competence.	FQ1: The information department will solve the problems I encounter in a timely manner. FQ2: The information department is able to understand and solve my problem with seriousness and patience. FQ3: The hospital provides adequate training in the use of information systems.	[28,44]
Use intention (US)	Users’ willingness to use information systems.	US1: I would like to use this information system. US2: I tend to use this information system to assist my work.	[35]
Satisfaction (SA)	Users’ level of satisfaction with system, information, and support services.	SA1: I am satisfied with the system quality of the information system. SA2: I am satisfied with the information quality of the information system. SA3: I am satisfied with the service quality of the information system. SA4: I am satisfied with the whole information system.	[44]
Individual benefits (communication [CB] and decision-making [DB])	The extent to which information systems are contributing to the success of individuals on their communication, collaboration, and decision-making.	By using the information system, I am able to: CB1: Communicate more effectively with colleagues. CB2: Communicate and collaborate more closely with colleagues. CB3: Communicate more directly with colleagues. CB4: Facilitate communication and collaboration between departments. DB1: Gather more comprehensive information for decision-making. DB2: Analyze more alternatives in decision-making. DB3: Make decisions faster. DB4: Improve decision-making quality.	[45]
Organization benefits (OB)	The extent to which information systems are contributing to the success of organizations on infection prevention and control (IPC) management.	OB1: The use of the information system allows management to capture changes in a timely manner (such as irrational antibiotic prescriptions, changes in hospital drug resistance trends, etc). OB2: The use of the information system helps the hospital to respond to the emergency of hospital infection more quickly. OB3: The use of the information system makes the management more efficient. OB4: The use of the information system helps hospitals save management costs of IPC.	[44]
IPC culture (OC)	The increasingly stable core values ​​and concepts gradually formed around improving IPC, as well as the environmental characteristics, rules and regulations, and group awareness derived on this basis.	OC1: Staff will freely speak up if they see something that may improve patient care or affect patient safety. OC2: People in this organization are comfortable to communicate with each other if they have questions about the right way to infection prevention. OC3: The healthcare-associated infection prevention goals and strategic plans of our organization are clear and well communicated. OC4: Employees are encouraged to become involved in infection prevention. OC5: Most people in this organization are so busy that they have very little time to devote to infection prevention efforts. OC6: I can think of examples when problems with patient infections have led to changes in our procedures or equipment.	[46]

#### Hypothesis

##### Influence of Technical Factors on Satisfaction and Use Intention

Different types of information system qualities (system, information, and service qualities) positively affect users’ satisfaction and use intention, based on the D&M model and a review [27,44,47]. For medical staff, Pai et al [48] proved that information, service, and system qualities influence use intention based on the technology acceptance model and D&M model. It was proven that information, service, and system qualities significantly improve the satisfaction of medical staff with hospital information systems based on the Unified Theory of Acceptance and Use Technology and the D&M model [49].

Therefore, this study hypothesizes the following:

- H1: Technical factors (system quality [H1a], information quality [H1b], and service quality [H1c]) have a positive impact on users’ satisfaction.
- H2: Technical factors (system quality [H2a], information quality [H2b], and service quality [H2c]) have a positive impact on users’ use intention.

##### Influence of IPC Culture on Use Intention

Previous information system studies have found that cultural values influence the acceptance of information technology (eg, [50,51]). In the health field, the organizational culture of a health care facility is an important factor in the successful implementation of information technology [52]. Research confirmed that organizational culture affects clinicians’ attitudes toward the use of clinical information systems. Queenan et al also verified that the interaction between patient safety culture and the computerized provider order entry (CPOE) system improves the ability of organizations to process information, which in turn promotes the success of CPOE system application [53]. Therefore, the following assumption was made:

- H3: The culture of IPC has a positive impact on the use of IPCISs.

##### Influence of Satisfaction on Use Intention

User satisfaction is considered to be one of the necessary factors for evaluating the success of information systems. Studies confirmed a direct and significant relationship between user satisfaction and system use intention [54]. Chow et al demonstrated that nurses tended to be more satisfied with a user-friendly system and were, therefore, more involved in its use [55]. Thus, the following assumption was made:

- H4: User satisfaction has a positive impact on the intention to use the system.

##### Influence of Use Intention and Satisfaction on Net Benefits

The D&M model’s net benefits include individual and organizational benefits, representing the degree to which the user/organization believes that the use of the system will bring benefits, such as improving the user’s or organization’s job performance, productivity, etc [56]. An increasing number of hospital practices have confirmed that using information systems improves workflow and improves medical service quality [57]. Hou et al confirmed that nurses using an electronic handover system can improve their communication efficiency [58]. Jalali et al discovered that information systems involving drug interactions can support physicians in making decisions more efficiently [59]. Literature evidence on the impact of satisfaction suggests that employees who are satisfied with a system are more likely to be more productive, especially if the use of such a system is mandated [60-62]. In a previous report [49], it was found that there was a significant positive correlation between medical satisfaction and personal influence, and in another report [63], clinicians’ satisfaction positively impacted the quality of clinical decision-making. Therefore, we hypothesize the following:

- H5: Use intention has a positive impact on individual benefits (decision-making [H5a] and communication [H5b]) and organizational benefits (H5c).
- H6: User satisfaction has a positive impact on individual benefits (decision-making [H6a] and communication [H6b]) and organizational benefits (H6c).

### Participants

Participants of the study were clinicians from 307 secondary and tertiary hospitals in Hubei Province, China. The inclusion criteria were as follows: clinicians with experience in using IPCISs, those who knew the purpose of the study, and those who were willing to cooperate. In addition, to ensure a balanced sample, we required a sample size of at least eight clinicians each (including at least two members of the hospital IPC team) in the departments of respiratory medicine, urology, intensive care, neurology, endocrinology, and orthopedics, and at least five clinicians each in other departments.

### Ethical Considerations

This study was approved by the ethics committee of Tongji Medical College, Huazhong University of Science and Technology (2021-S063). All participants provided written informed consent, which included survey purpose, survey period, privacy protection, and investigator information. The study data were anonymized and deidentified.

### Instrument

This survey designed the constructs of information system quality (system, information, and service quality), use intention, satisfaction, individual benefits, and organizational benefits based on the D&M model, and added the construct of IPC culture based on the organizational culture perspective. The measurement items we used were adapted from previous related studies with modifications to suit the context of the study. To ensure the validity of the content, we asked 6 experts (2 from the information management field, 2 from hospital management, and 2 from the clinical front line) to check the clarity, redundancy, and understandability of the survey entries (see Table 1 for the constructs and measures). In addition, we tested the reliability and validity of the questionnaire. Kaiser-Meyer-Olkin (KMO) and Bartlett sphericity tests were performed on the constructs. The closer the KMO value is to 1, the stronger is the correlation between the constructs and the more suitable they are for factor analysis. The Cronbach α coefficient was used to evaluate the reliability of internal consistency among items, and the overall Cronbach α coefficient of the questionnaire was generally required to be above .7. In this study, the reflective items were screened through exploratory factor analysis and confirmatory factor analysis, and a questionnaire with reliability and validity was obtained. The final questionnaire comprised 7 components and 41 measurement items, including demographic characteristics, technical factors, hospital IPC culture, use intention, satisfaction, individual benefits, and organizational benefits. Scoring was performed using a 5-point Likert scale (1, completely disagree; 2, disagree; 3, not sure; 4, agree; 5, strongly agree). We used adaptive questioning and simplified the expression of the items to reduce the complexity of the questionnaire.

### Data Collection

The questionnaire was generated through an online questionnaire platform [64] and distributed through an electronic link. Prior to distribution, researchers conducted predictive tests to ensure the online questionnaire’s availability and technical functionality, ensuring no vulnerabilities were present. The questionnaire was exclusively open to clinical doctors within the hospital information platform. To prevent participation from nontargeted visitors, an initial termination question was included in the questionnaire (ie, “Are you a clinician or nonclinician?”). If a respondent selected “nonclinician,” their questionnaire response was immediately terminated. The platform determined unique visitors based on the user IP address and user login name. Questionnaire response was mandatory and was supported by the government, which can avoid volunteer bias. Following the strategy of “protecting respondents’ anonymity and reducing assessment anxiety,” we assured respondents that their responses are anonymous and emphasized that there are no right or wrong answers. Finally, we arranged for 2 researchers to check the consistency and completeness of the questionnaire. Data collection lasted for a week.

### Data Analysis

Partial least squares-structural equation modeling (PLS-SEM) was used to analyze the extended D&M model in this study by using the SmartPLS software (v3.39) [65]. PLS-SEM is a variance-based approach estimating the parameters of a set of equations in a structural equation model by combining principal component analysis with regression-based path analysis [66]. It can handle complex models; has fewer limitations on data distribution, variable type, and actual sample size; and is suitable for developmental theories [67]. The reasons for using PLS-SEM were that the relevant data in this study were not normally distributed, and the extended D&M model was exploratory with a large number of structural model relationships. In order to further understand the influence degrees of different levels in constructs and their measurement items, this study conducted an importance-performance matrix analysis (IPMA). The IPMA results allowed prioritization of constructs and their measurement items to identify the most important factors for improvement. Typically, factors with relatively high importance and relatively low performance require the most attention [68,69].

## Results

### Demographic Characteristics of the Clinicians

A total of 12,493 clinicians from 246 secondary and tertiary medical institutions answered the questionnaire, and 12,317 questionnaires met the requirements after review, with a response rate of 98.6%. The demographic characteristics of the clinicians surveyed are shown in Table 2.

**Table 2:** Demographic characteristics of the clinicians.

Characteristic		Value (N=12,317), n (%)
Gender (male)		6535 (53.1)
**Hospital type**		
	Tertiary hospital	7917 (64.3)
	Secondary hospital	4400 (35.7)
**Title**		
	Chief	614 (5.0)
	Associate chief	2474 (20.1)
	Doctor	5289 (42.9)
	Assistant doctor	3448 (28.0)

### Evaluation of Measurement Models

Composite reliability and Cronbach α values were used to assess the internal reliability of reflective constructs. The results showed that all composite reliability values were higher than the recommended value of 0.8 (range: 0.934-0.992), and Cronbach α values were above the recommended value of .7, indicating sufficient reliability for all constructs. The results for convergent validity showed that the average variance extracted of all constructs was greater than the recommended threshold of 0.5 (range: 0.806-0.967), suggesting that the variance of all constructs was greater than the variation induced by individual measurement error, which confirmed the convergence validity (Table 3). The discriminant validity involved the Fornell and Larcker criterion. The results in Multimedia Appendix 1 demonstrate that the root mean square for each construct is higher than the cross-loading.

**Table 3:** Construct reliability and validity.

Construct	Cronbach α	rho_A	Composite reliability	Average variance extracted
System quality	.915	0.919	0.946	0.854
Information quality	.979	0.979	0.987	0.961
Service quality	.963	0.963	0.976	0.931
IPC^a^ culture	.952	0.952	0.961	0.806
Satisfaction	.986	0.986	0.989	0.959
Use intention	.859	0.870	0.934	0.876
Decision-making	.989	0.989	0.992	0.967
Communication	.987	0.987	0.990	0.950
Organizational benefits	.991	0.991	0.992	0.956

^a^IPC: infection prevention and control.

### Evaluation of the Structural Model

After confirming the reliability and validity of the measurement model, we tested the hypotheses by estimating the structural model. The explanatory power of the model was evaluated with the *R*^2^ score. The *R*^2^ values of satisfaction (0.833), use intention (0.821), individual benefits in communication (0.624), individual benefits in decision-making (0.676), and organization benefits (0.656) were all higher than the level of 0.5. This indicates that the extended D&M model had a moderate to high level of explanatory power, accounting for 82.1% of the variance in use intention. Evaluating the effect size (f^2^), it was found that the impact size of system use on individual decision-making benefits was large (2.090).

Through the bootstrapping process, we provided normalized path coefficients for each path to compare the effects of different constructs. First, we found that users’ use intention was significantly related to individual decision-making benefits (β=.474; *P*<.001), individual communication benefits (β=.466; *P*<.001), and organizational benefits (β=.461; *P*<.001), which suggests that when individuals are willing to use IPCISs to support their IPC work, they are better able to communicate with each other and make decisions, as well as facilitate management at the organizational level. Therefore, the hypotheses H6a, H6b, and H6c are supported.

**Figure 2:**
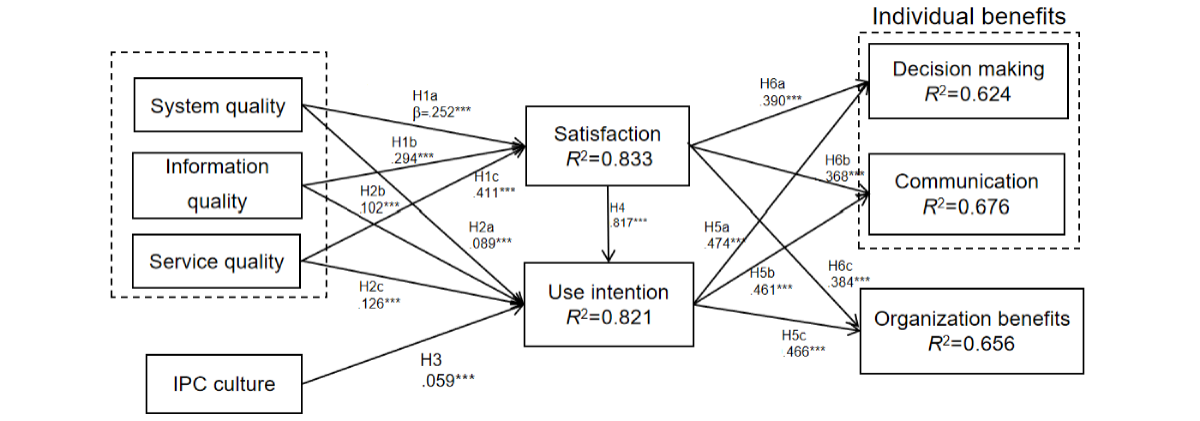
Results of the structural model. The hypotheses are as follows: H1: Technical factors (system quality [H1a], information quality [H1b], and service quality [H1c]) have a positive impact on users’ satisfaction; H2: Technical factors (system quality [H2a], information quality [H2b], and service quality [H2c]) have a positive impact on users’ use intention; H3: The culture of IPC has a positive impact on the use of IPCISs; H4: User satisfaction has a positive impact on the intention to use the system; H5: Use intention has a positive impact on individual benefits (decision-making [H5a] and communication [H5b]) and organizational benefits (H5c); H6: User satisfaction has a positive impact on individual benefits (decision-making [H6a] and communication [H6b]) and organizational benefits (H6c). IPC: infection prevention and control; IPCIS: infection prevention and control information system. ***P<.001.

After understanding the effects of use intention and satisfaction, we further investigated the antecedents that may drive individuals to use IPCISs and their satisfaction. The results showed that all quality dimensions (system quality, information quality, and service quality) had a significant effect on user satisfaction (system quality: β=.252; *P*<.001; information quality: β=.294; *P*<.001; service quality: β=.411; *P*<.001). Therefore, our data support hypotheses H1a, H1b, and H1c. With regard to use intention, there was significant influence by system quality (β=.089; *P*<.001), information quality (β=.102; *P*<.001), and service quality (β=.125; *P*<.001). Moreover, IPC culture (β=.059; *P*<.001) had a significant impact on use intention. Thus, hypotheses H2a, H2b, H2c, and H3 are supported. The structural model is shown in Figure 2. These results suggest that in addition to technical factors, organizational culture in IPC is an important influencing factor of use intention and satisfaction (Table 4).

**Table 4:** Assessment of the structural model.

Variable	Standard coefficient (β)	T statistics (|O/STDEV|^a^)	2.50% value	97.50% value	Statistical significance
Use intention → decision making	.474	24.005	0.436	0.513	Yes
Use intention → organization benefits	.466	23.799	0.429	0.504	Yes
Use intention → communication	.461	22.310	0.423	0.503	Yes
Satisfaction → decision making	.390	19.884	0.349	0.426	Yes
Satisfaction → communication	.368	17.838	0.328	0.403	Yes
Satisfaction → organization benefits	.384	19.561	0.344	0.420	Yes
Satisfaction → use intention	.581	37.460	0.554	0.611	Yes
Service quality → satisfaction	.411	26.197	0.382	0.443	Yes
Information quality → satisfaction	.294	16.849	0.260	0.327	Yes
System quality → satisfaction	.252	18.396	0.226	0.277	Yes
Service quality → use intention	.125	7.672	0.092	0.155	Yes
Information quality → use intention	.102	5.927	0.066	0.137	Yes
System quality → use intention	.089	6.964	0.063	0.113	Yes
IPC^b^ culture → use intention	.059	8.394	0.045	0.073	Yes

^a^|O/STDEV|: the absolute value of the observed statistic divided by the standard deviation.

^b^IPC: infection prevention and control.

### IPMA Results

**Figure 3:**
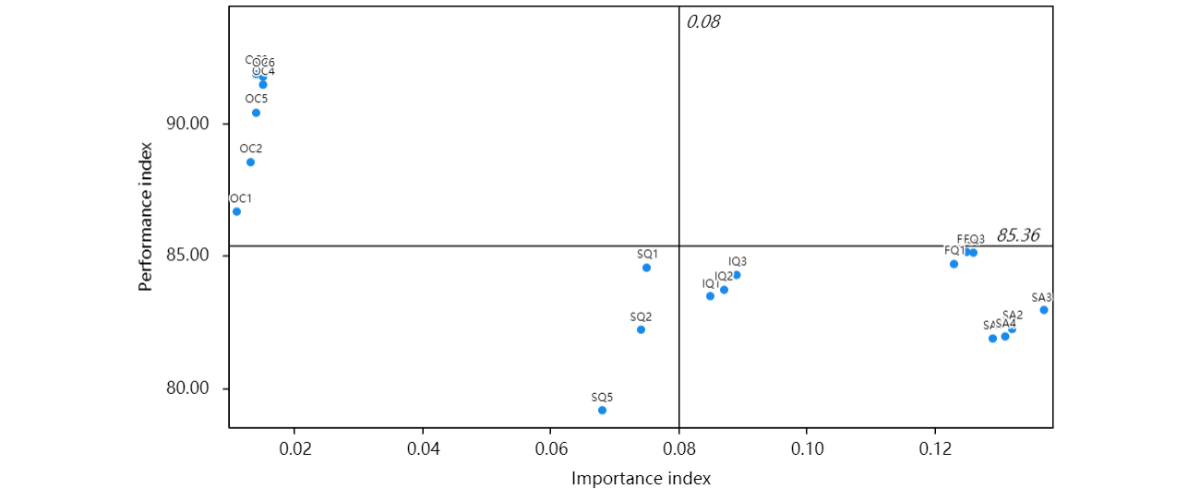
The importance-performance matrix analysis results of use intention. The measurement items are presented in Table 1.

It was found from the path coefficient that use intention had a greater impact on net benefits; thus, this study focused on the prioritization of attribute-level factors that affect use intention. We have shown the importance and performance indices of construct attributes in a grid (Figure 3), and the attributes present in the fourth quadrant are the ones that need to be improved as they represent high relative importance but low relative performance. We found that satisfaction, service quality, and information quality had higher importance and lower performance regarding use intention. In particular, the satisfaction index SA3 (satisfaction with the service quality of the information system), the service quality index FQ3 (the hospital provides training in the use of the information system), and the information quality index IQ3 (the legibility of the information) are directions that can be further improved. Additional assessment results of IPC culture are provided in Multimedia Appendix 2.

## Discussion

### Principal Findings

This study evaluated the application of IPCISs from the perspective of clinicians, using the extended D&M model as the theoretical framework. The extended model incorporates the cultural construct, providing a more comprehensive evaluation of IPCISs. Consistent with previous studies [70,71], our findings indicate that the technical factors of IPCISs, namely system quality, information quality, and service quality, positively impact user satisfaction and use intention. In addition, our research confirms that the use intention of IPCISs has an impact on individuals’ decision-making and communication, and organizational effectiveness, such as organizational management and response to hospital infection emergencies. This is partially consistent with similar findings in a previous report [72]. Notably, our study revealed that system usage contributes more to net benefits than satisfaction. This implies that if IPCISs meet the clinicians’ needs, they will experience the benefits of using the systems. Through this result, it can be seen that the application of information systems has a significant effect on the benefits that individuals and organizations are most concerned about. Therefore, it is crucial for hospitals to focus on system improvements that align with the actual needs of users, enabling effective decision-making and communication in IPC, as well as organizational IPC management.

Our research offers new insights that differ from the insights of previous studies. In contrast to prior research [70,71], our IPMA revealed that service quality is a more important measure of the willingness to use IPCISs, followed by information quality. Among them, the dimension FQ3 (the hospital provides sufficient training on system use) emerged as a crucial component indicator for service quality. This highlights the necessity of enhancing clinicians’ operational capabilities when using IPCISs, as advocated by the APIC. Hospitals should consider augmenting training support to enhance clinicians’ proficiency in system usage. Research [73] also mentioned that providing end-user training and management support helps to create a supportive environment, which will pay dividends in increasing the use and effectiveness of the system. Regarding information quality, our analysis demonstrated that clinicians placed a premium on easy-to-read information fields and reports (dimension IQ3). Relevant research findings also confirmed that the information presentation method of the experimental inspection report impacts clinical treatment efficiency and drug use [74]. Bauer et al found that improving the presentation of biochemical metric reports allowed physicians to achieve better results in terms of assessment speed and user satisfaction [75]. Consequently, the findings derived from the IPMA provide specific directions for further improving IPCISs.

It is worth noting that this study found a significant positive influence of IPC culture on use intention, which is consistent with similar findings in previous research [52,53]. However, the IPMA results indicate that IPC culture has a relatively lower importance compared to other factors in influencing the intention to use information systems. We believe this reflects the current lack of emphasis on using information technology to assist in hospital infection control, despite the significant impact of IPC culture on the intention to use information systems. This highlights the significant interaction between organizational culture and use intention in task-oriented organizations [76], and indicates the need for interventions from IPC culture to enhance the awareness and information abilities of clinicians. Research indicates that besides the factors related to system usability, numerous organizational and cultural factors also affect the use of hospital management information systems [72]. Callen et al discovered that in constructive hospital cultures, health care professionals exhibited a positive attitude toward information systems, while in defensive and negative cultures, individuals display a negative attitude toward information systems [52]. Organizational culture, as a crucial social characteristic within an organization, is often overlooked when implementing information technologies. Solely emphasizing system performance may not lead to effective implementation. Our research findings indicate that strengthening the cultural atmosphere of IPC within hospitals can increase health care professionals’ attention to IPC and promote the use of IPCISs. Currently, countries worldwide are designing and implementing measures for hospital IPC, and our research results can provide evidence-based support for intervention measures.

### Limitations

Although this study provides interesting insights into understanding the effects of IPCIS applications, there are some limitations to consider. First, given the cross-sectional survey used to collect data in this study, our finding about how technical factors and hospital IPC culture affect IPCIS use cannot fully reveal the causal relationship between variables. Second, the sample for this study comes from a certain region. Although the sample size was quite large and representative, the generalizability of the research findings needs to be verified. In the future, longitudinal studies could be conducted, with collection of data in multiple stages to verify causality. Additionally, comparative studies on the use and evaluation of IPCISs in hospitals regarding different levels and types could be added to better provide insights for IPCIS construction.

### Conclusions

This study evaluated the application of IPCISs among clinicians based on the extended D&M model. We confirmed the applicability of the extended D&M model in studying the use of IPCISs. We found that technical factors (system quality, information quality, and service quality) and hospital IPC culture have an impact on the successful use of IPCISs. Among them, service quality and information quality showed higher importance and lower performance for use intention. Moreover, use intention had a greater impact on decision-making in net benefits. Furthermore, the results of the IPMA suggested that providing information system training and improving the legibility of information in the system could improve the use intention of clinicians. These findings provide empirical evidence and specific practical directions for further improving the construction of IPCISs. We suggest that hospitals conduct more training on the use of IPCISs, modify the information presentation form on the system, and adjust the amount of information to improve the efficient use of IPC information by clinicians.

## Appendix

Multimedia Appendix 1 Discriminant validity.

Multimedia Appendix 2 Additional assessment results of infection prevention and control culture.

## Abbreviations

APIC: Association for Professionals in Infection Control and Epidemiology
CPOE: computerized provider order entry
D&M model: DeLone and McLean Information Systems Success model
HAI: healthcare-associated infections
IPC: infection prevention and control
IPCIS: infection prevention and control information system
IPMA: importance-performance matrix analysis
KMO: Kaiser-Meyer-Olkin
PLS-SEM: partial least squares-structural equation modeling

## Data Availability

The data sets generated during and/or analyzed during this study are available from the corresponding author on reasonable request.

## References

[1] Sievert DM, Ricks P, Edwards JR, Schneider A, Patel J, Srinivasan A, Kallen A, Limbago B, Fridkin S, National Healthcare Safety Network (NHSN) TeamParticipating NHSN Facilities (2013). Antimicrobial-resistant pathogens associated with healthcare-associated infections: summary of data reported to the National Healthcare Safety Network at the Centers for Disease Control and Prevention, 2009-2010. Infect Control Hosp Epidemiol.

[2] Guckian J, Jobling K, Oliphant T, Weatherhead S, Blasdale K (2020). 'I saw it on Facebook!' Assessing the influence of social media on patient presentation to a melanoma screening clinic. Clin Exp Dermatol.

[3] Gilbert G, Kerridge I (2020). Hospital infection control: old problem - evolving challenges. Intern Med J.

[4] Dettenkofer M, Humphreys H, Saenz H, Carlet J, Hanberger H, Ruef C, Widmer A, Wolkewitz M, Cookson B (2016). Key priorities in the prevention and control of healthcare-associated infection: a survey of European and other international infection prevention experts. Infection.

[5] Reilly J, McCoubrey J, Cole S, Khan A, Cook B (2015). Integrating intensive care unit (ICU) surveillance into an ICU clinical care electronic system. J Hosp Infect.

[6] Schönfeld V, Diercke M, Gilsdorf A, Eckmanns T, Walter J (2018). Evaluation of the statutory surveillance system for invasive MRSA infections in Germany, 2016-2017. BMC Public Health.

[7] (2001). Guidelines on prevention and control of hospital associated infections : report of an informal consultation, Bangkok, Thailand, 26-29 June 2001. World Health Organization.

[8] Komasawa M, Aung MN, Saito K, Isono M, Tanaka G, Makimoto S (2021). Overcoming Current and Preventing Future Nosocomial Outbreaks during the COVID-19 Pandemic: Lessons Learned at Three Hospitals in Japan. Int J Environ Res Public Health.

[9] Noaman AY, Ragab AHM, Al‐Abdullah N, Jamjoom A, Nadeem F, Ali AG (2019). Predicting and reducing “hospital‐acquired infections” using a knowledge‐based e‐surveillance system. Expert Systems.

[10] Durand C, Alfandari S, Béraud G, Tsopra R, Lescure F, Peiffer-Smadja N (2022). Clinical Decision Support Systems for Antibiotic Prescribing: An Inventory of Current French Language Tools. Antibiotics (Basel).

[11] Kaur J, Kaur J, Kapoor S, Singh H (2021). Sci Rep.

[12] Park J, Seale H (2017). Examining the online approaches used by hospitals in Sydney, Australia to inform patients about healthcare associated infections and infection prevention strategies. BMC Infect Dis.

[13] Tilahun B, Fritz F (2015). Service Quality: A Main Determinant Factor for Health Information System Success in Low-resource Settings. Stud Health Technol Inform.

[14] Sax H, Schreiber PW, Clack L, Ratz D, Saint S, Greene MT, Kuster SP (2020). Preventing healthcare-associated infection in Switzerland: Results of a national survey. Infect Control Hosp Epidemiol.

[15] Falkenberg E, Hesse W, Lindgreen P, Nilsson B, Oei J, Rolland C, Stamper R, van Assche F, Verrijn-Stuart A, Voss K (1998). FRISCO: A framework of information system concepts : The FRISCO report (WEB edition).

[16] Aghdassi SJS, Kohlmorgen B, Schröder C, Peña Diaz LA, Thoma N, Rohde AM, Piening B, Gastmeier P, Behnke M (2021). Implementation of an automated cluster alert system into the routine work of infection control and hospital epidemiology: experiences from a tertiary care university hospital. BMC Infect Dis.

[17] Avina K, Sinha RK (2022). Development of an Automated Hospital Infection Control Surveillance Toolkit. Journal of Health Management.

[18] Verberk J, Aghdassi S, Abbas M, Nauclér P, Gubbels S, Maldonado N, Palacios-Baena Z, Johansson A, Gastmeier P, Behnke M, van Rooden S, van Mourik M (2022). Automated surveillance systems for healthcare-associated infections: results from a European survey and experiences from real-life utilization. J Hosp Infect.

[19] Simões AS, Maia M, Gregório J, Couto I, Asfeldt A, Simonsen G, Póvoa P, Viveiros M, Lapão LV (2018). Participatory implementation of an antibiotic stewardship programme supported by an innovative surveillance and clinical decision-support system. J Hosp Infect.

[20] Tsutsui A, Suzuki S (2018). Japan nosocomial infections surveillance (JANIS): a model of sustainable national antimicrobial resistance surveillance based on hospital diagnostic microbiology laboratories. BMC Health Serv Res.

[21] Iversen A, Kavalaris CP, Hansen R, Hansen MB, Alexander R, Kostadinov K, Holt J, Kristensen B, Knudsen JD, Møller JK, Ellermann-Eriksen S (2020). Clinical experiences with a new system for automated hand hygiene monitoring: A prospective observational study. Am J Infect Control.

[22] Knudsen A, Kolle S, Hansen M, Møller JK (2021). Effectiveness of an electronic hand hygiene monitoring system in increasing compliance and reducing healthcare-associated infections. J Hosp Infect.

[23] Behnke M, Valik JK, Gubbels S, Teixeira D, Kristensen B, Abbas M, van Rooden SM, Gastmeier P, van Mourik MS, PRAISE network (2021). Information technology aspects of large-scale implementation of automated surveillance of healthcare-associated infections. Clin Microbiol Infect.

[24] van Mourik MSM, Perencevich E, Gastmeier P, Bonten M (2018). Designing Surveillance of Healthcare-Associated Infections in the Era of Automation and Reporting Mandates. Clin Infect Dis.

[25] van Mourik MS, van Rooden SM, Abbas M, Aspevall O, Astagneau P, Bonten MJ, Carrara E, Gomila-Grange A, de Greeff SC, Gubbels S, Harrison W, Humphreys H, Johansson A, Koek MB, Kristensen B, Lepape A, Lucet J, Mookerjee S, Naucler P, Palacios-Baena ZR, Presterl E, Pujol M, Reilly J, Roberts C, Tacconelli E, Teixeira D, Tängdén T, Valik JK, Behnke M, Gastmeier P, PRAISE network (2021). PRAISE: providing a roadmap for automated infection surveillance in Europe. Clin Microbiol Infect.

[26] Deryabina A, Lyman M, Yee D, Gelieshvilli M, Sanodze L, Madzgarashvili L, Weiss J, Kilpatrick C, Rabkin M, Skaggs B, Kolwaite A (2021). Core components of infection prevention and control programs at the facility level in Georgia: key challenges and opportunities. Antimicrob Resist Infect Control.

[27] DeLone WH, McLean ER (1992). Information Systems Success: The Quest for the Dependent Variable. Information Systems Research.

[28] Delone WH, McLean ER (2014). The DeLone and McLean Model of Information Systems Success: A Ten-Year Update. Journal of Management Information Systems.

[29] Tam C, Oliveira T (2016). Computers in Human Behavior.

[30] Widjaja A, Chen J, Gonchig B (2018). Investigating Factors Affecting Central Bank Information Systems Success: The Case of the Central Bank of Mongolia. International Journal of Technology and Human Interaction.

[31] Alshardan A, Goodwin R, Rampersad G (2015). A Benefits Assessment Model of Information Systems for Small Organizations in Developing Countries. CIS.

[32] Alzahrani AI, Mahmud I, Ramayah T, Alfarraj O, Alalwan N (2017). Modelling digital library success using the DeLone and McLean information system success model. Journal of Librarianship and Information Science.

[33] Noudoostbeni A, Kaur K, Jenatabadi H (2018). Sustainability.

[34] Akrong G, Shao Y, Owusu E (2021). Assessing the Impact of System Quality, Information Quality, and Service Quality on Enterprise Resource Planning (ERP) Systems. International Journal of Enterprise Information Systems.

[35] Hsu P, Yen HR, Chung J (2015). Assessing ERP post-implementation success at the individual level: Revisiting the role of service quality. Information & Management.

[36] Van Der Meijden MJ, Tange HJ, Troost J, Hasman A (2003). Determinants of success of inpatient clinical information systems: a literature review. J Am Med Inform Assoc.

[37] Yu P, Qian S (2018). Developing a theoretical model and questionnaire survey instrument to measure the success of electronic health records in residential aged care. PLoS One.

[38] Tschannen D, Talsma A, Reinemeyer N, Belt C, Schoville R (2011). Nursing Medication Administration and Workflow Using Computerized Physician Order Entry. CIN: Computers, Informatics, Nursing.

[39] Detert JR, Schroeder RG, Mauriel JJ (2000). A Framework for Linking Culture and Improvement Initiatives in Organizations. The Academy of Management Review.

[40] Leidner D, Kayworth T (2006). Review: A Review of Culture in Information Systems Research: Toward a Theory of Information Technology Culture Conflict. MIS Quarterly.

[41] Vogus T, Sutcliffe K (2007). The Safety Organizing Scale: development and validation of a behavioral measure of safety culture in hospital nursing units. Med Care.

[42] Hanchett M APIC’s new tool serves as a road map for personalized, professional career growth. APIC.

[43] Zheng F, Wang K, Wang Q, Yu T, Zhang X (2023). The pre-analytical process management status and influencing factors of laboratory test before prescribing antimicrobial in developing country. BMC Health Serv Res.

[44] Petter S, DeLone W, McLean E (2017). Measuring information systems success: models, dimensions, measures, and interrelationships. European Journal of Information Systems.

[45] Wang Y, Wang H, Shee DY (2007). Measuring e-learning systems success in an organizational context: Scale development and validation. Computers in Human Behavior.

[46] Colet PC, Cruz JP, Cacho G, Al-Qubeilat H, Soriano SS, Cruz CP (2018). Perceived Infection Prevention Climate and Its Predictors Among Nurses in Saudi Arabia. J Nurs Scholarsh.

[47] Gaardboe R, Sandalgaard N, Nyvang T (2022). An assessment of business intelligence in public hospitals. IJISPM.

[48] Pai F, Huang K (2011). Applying the Technology Acceptance Model to the introduction of healthcare information systems. Technological Forecasting and Social Change.

[49] Kuo K, Liu C, Talley PC, Pan S (2018). Strategic Improvement for Quality and Satisfaction of Hospital Information Systems. J Healthc Eng.

[50] Hogan SJ, Coote LV (2014). Organizational culture, innovation, and performance: A test of Schein's model. Journal of Business Research.

[51] Metallo C, Agrifoglio R, Lepore L, Landriani L (2022). Explaing users' technology acceptance through national cultural values in the hospital context. BMC Health Serv Res.

[52] Callen JL, Braithwaite J, Westbrook JI (2007). Cultures in hospitals and their influence on attitudes to, and satisfaction with, the use of clinical information systems. Soc Sci Med.

[53] Queenan CC, Kull TJ, Devaraj S (2016). Complements or Substitutes? Culture-Technology Interactions in Healthcare. Decision Sciences.

[54] Balaban I, Mu E, Divjak B (2013). Development of an electronic Portfolio system success model: An information systems approach. Computers & Education.

[55] Chow S, Chin WY, Lee HY, Leung HC, Tang FH (2012). Nurses' perceptions and attitudes towards computerisation in a private hospital. J Clin Nurs.

[56] Staples D, Wong I, Seddon PB (2002). Having expectations of information systems benefits that match received benefits: does it really matter?. Information & Management.

[57] van de Wetering R (2018). IT-Enabled Clinical Decision Support: An Empirical Study on Antecedents and Mechanisms. J Healthc Eng.

[58] Hou Y, Lu L, Lee P, Chang I (2019). Positive Impacts of Electronic hand-off systems designs on Nurses' communication effectiveness. J Nurs Manag.

[59] Jalali A, Johannesson P, Perjons E, Askfors Y, Kalladj AR, Shemeikka T, Vég A (2019). Evaluating a Clinical Decision Support System for Drug-Drug Interactions. Stud Health Technol Inform.

[60] Holsapple CW, Wang Y, Wu J (2005). Empirically Testing User Characteristics and Fitness Factors in Enterprise Resource Planning Success. International Journal of Human-Computer Interaction.

[61] Lwoga ET, Sangeda RZ, Mushi R (2020). Predictors of electronic health management information system for improving the quality of care for women and people with disabilities. Information Development.

[62] Muin H, Palutturi S, Sirajuddin S, Mallongi A, Syam A (2020). User's satisfaction about the use of simrs performance of outpatient units in nene mallomo hospital sidenreng rappang regency. Enfermería Clínica.

[63] Shen C, Chang R, Hsu CJ, Chang I (2017). How business intelligence maturity enabling hospital agility. Telematics and Informatics.

[64] WJX.

[65] Hair J, Hollingsworth CL, Randolph AB, Chong AYL (2017). An updated and expanded assessment of PLS-SEM in information systems research. IMDS.

[66] Sarstedt M, Ringle C, Hair J, Homburg C, Klarmann M, Vomberg A (2021). Partial Least Squares Structural Equation Modeling. Handbook of Market Research.

[67] Fong L, Law R (2013). Hair, J. F. Jr., Hult, G. T. M., Ringle, C. M., Sarstedt, M. (2014). A Primer on Partial Least Squares Structural Equation Modeling (PLS-SEM). Sage Publications. ISBN: 978-1-4522-1744-4. 307 pp. EJTR.

[68] Ahmad S, Afthanorhan WMABW (2014). The Importance-Performance Matrix Analysis in Partial Least Square Structural Equation Modeling (PLS-SEM) with Smartpls 2.0 M3. 24.

[69] Ting SH, Yahya S, Tan CL (2019). Importance-Performance Matrix Analysis of the Researcher’s Competence in the Formation of University-Industry Collaboration Using Smart PLS. Public Organiz Rev.

[70] Çelik K, Ayaz A (2021). Validation of the Delone and McLean information systems success model: a study on student information system. Educ Inf Technol.

[71] Petter S, Fruhling A (2011). Evaluating the success of an emergency response medical information system. Int J Med Inform.

[72] Kivinen T, Lammintakanen J (2013). The success of a management information system in health care - a case study from Finland. Int J Med Inform.

[73] Igbaria M (1990). End-user computing effectiveness: A structural equation model. Omega.

[74] Torsvik T, Lillebo B, Mikkelsen G (2013). Presentation of clinical laboratory results: an experimental comparison of four visualization techniques. J Am Med Inform Assoc.

[75] Bauer DT, Guerlain S, Brown PJ (2010). The design and evaluation of a graphical display for laboratory data. J Am Med Inform Assoc.

[76] Kanungo S (1998). An empirical study of organizational culture and network-based computer use. Computers in Human Behavior.

