# Supplementary material for: Factors Influencing Clinicians’ Use of Hospital Information Systems for Infection Prevention and Control: Cross-Sectional Study Based on the Extended DeLone and McLean Model
Source: J Med Internet Res. 2023 Jun 22;25:e44900. doi: 10.2196/44900 (PMC10337337; doi:10.2196/44900)
Supplement: Multimedia Appendix 1 [file jmir_v25i1e44900_app1.docx]

**Multimedia Appendix 1.** Discriminant validity.

|  | Service quality | Communication | Information quality | Decision making | IPC^a^ culture | Satisfaction | System quality | Use intention | Organizational benefits |
| --- | --- | --- | --- | --- | --- | --- | --- | --- | --- |
| Service quality | 0.965 |  |  |  |  |  |  |  |  |
| Communication | 0.811 | 0.975 |  |  |  |  |  |  |  |
| Information quality | 0.883 | 0.780 | 0.980 |  |  |  |  |  |  |
| Decision making | 0.824 | 0.937 | 0.804 | 0.983 |  |  |  |  |  |
| IPC culture | 0.694 | 0.727 | 0.663 | 0.705 | 0.898 |  |  |  |  |
| Satisfaction | 0.880 | 0.780 | 0.876 | 0.813 | 0.635 | 0.979 |  |  |  |
| System quality | 0.834 | 0.742 | 0.873 | 0.773 | 0.643 | 0.851 | 0.924 |  |  |
| Use intention | 0.842 | 0.790 | 0.839 | 0.822 | 0.640 | 0.894 | 0.815 | 0.936 |  |
| Organizational benefits | 0.830 | 0.911 | 0.804 | 0.899 | 0.718 | 0.801 | 0.762 | 0.810 | 0.978 |

^a^IPC: infection prevention and control.
