# Supplementary material for: Factors Influencing Clinicians’ Use of Hospital Information Systems for Infection Prevention and Control: Cross-Sectional Study Based on the Extended DeLone and McLean Model
Source: J Med Internet Res. 2023 Jun 22;25:e44900. doi: 10.2196/44900 (PMC10337337; doi:10.2196/44900)
Supplement: Multimedia Appendix 2 [file jmir_v25i1e44900_app2.docx]

**Multimedia Appendix 2.** Additional assessment results of infection prevention and control culture.

Table S1. Factor loading Analysis of IPC culture.

| Variable | Indicator | loading factor |
| --- | --- | --- |
| IPC culture | OC1 | 0.835 |
|  | OC2 | 0.893 |
|  | OC3 | 0.889 |
|  | OC4 | 0.921 |
|  | OC5 | 0.933 |
|  | OC6 | 0.912 |

Table S2. IPC culture’s Item/Construct level importance and performance index.

|  | Item/Construct | Importance index | Performance index |
| --- | --- | --- | --- |
| Intention to use | OC1 | 0.011 | 86.661 |
|  | OC2 | 0.013 | 88.528 |
|  | OC3 | 0.014 | 91.859 |
|  | OC4 | 0.015 | 91.459 |
|  | OC5 | 0.014 | 90.397 |
|  | OC6 | 0.015 | 91.759 |
|  | OC | 0.081 | 90.273 |

The structural equation model diagram of the original model without IPC culture is as follow:


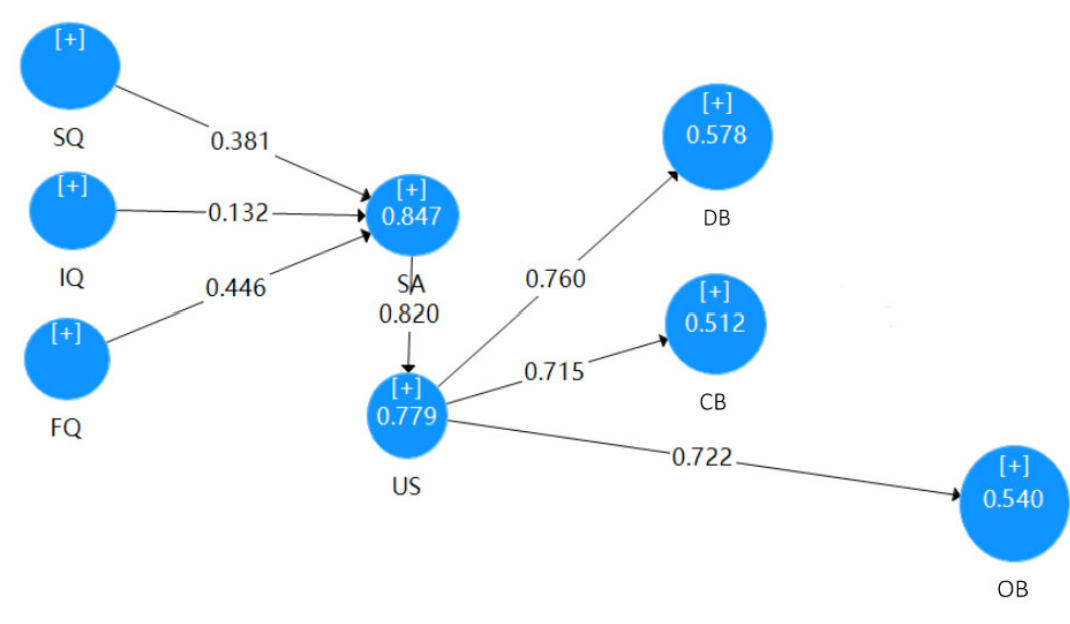


Figure S1. Results of structural model.
